# Retention of different temporary cements tested on zirconia crowns and titanium abutments in vitro

**DOI:** 10.1186/s40729-021-00349-4

**Published:** 2021-07-20

**Authors:** Felix Dähne, Heike Meißner, Klaus Böning, Christin Arnold, Ralf Gutwald, Elisabeth Prause

**Affiliations:** 1 Department of Oral and Plastic Maxillofacial Surgery, University Hospital Halle, Martin Luther University Halle-Wittenberg, Ernst-Grube-Str. 40, 06120 Halle, Germany; 2 Department of Prosthodontics, Carl Gustav Carus Faculty of Medicine, University of Technology, Fetscherstrasse 74, 01307 Dresden, Germany; 3 Department of Prosthodontics, School of Dental Medicine, Martin-Luther-University, Magdeburger Str. 16, 06112 Halle, Germany; 4 Faculty of Medicine/Dentistry, Danube Private University (DPU), Steiner Landstraße 124, 3500 Krems-Stein, Austria; 5 Department of Prosthodontics, Geriatric Dentistry and Craniomandibular Disorders, University Charité Berlin, Aßmannshauser Str. 4-6, 14197 Berlin, Germany

**Keywords:** Implantology, Zirconia crowns, Titanium, Retention, Temporary cements, Fracture mode analysis

## Abstract

**Purpose:**

The aim of the present study was to examine the retention force of monolithic zirconia copings cemented with various temporary cements on implant abutments in vitro.

**Methods:**

Sixty exercise implants with pre-screwed implant abutments were embedded in resin. Subsequently, 60 CAD/CAM manufactured zirconia copings were divided into three main groups [Harvard Implant Semi-permanent (HAV), implantlink semi Forte (IMP), Temp Bond NE (TBNE)]. The zirconia copings were cemented on the implant abutments and loaded with 35 N. Specimens were stored in distilled water (37 °C) for 24 h. Half of the test specimens of each group were subjected to a thermocycling (TC) process. Retention force was measured in a universal testing machine. Using magnifying glasses, the fracture mode was determined. Statistical analysis was performed applying the Kruskal-Wallis test, the post hoc test according to Dunn-Bonferroni and a chi-square test of independence.

**Results:**

Without TC, IMP showed the highest retention of the three temporary luting agents (100.5 ± 39.14 N). The measured retention forces of IMP were higher than those of HAV (45.78 ± 15.66 N) and TBNE (61.16 ± 20.19 N). After TC, retention was reduced. IMP showed the greatest retentive strength (21.69 ± 13.61 N, three fail outs). HAV and TBNE showed pull-off forces of similar magnitude (17.38 ± 12.77 N and 16.97 ± 12.36 N, two fail outs). The fracture mode analysis showed different results regarding the tested cements before and after TC (facture type before/after TC): IMP (III+II/III), HAV (I/II) and TBNE (III/III). There were clear differences of the fracture modes regarding the examination before and after TC.

**Conclusions:**

Within the limits of this study, IMP showed the highest pull-off forces under the chosen test conditions. All three temporary luting agents showed lower retention forces after TC. Retention values in the individual cement classes were very heterogeneous. Easy cement removal in the crown lumen favours the dominance of adhesive cement fractures on the abutment and adhesive/cohesive cement fractures on the abutment with HAV appears advantageous in case of recementation of the superstructure.

## Background

Implant-supported superstructures can be screw or cement retained. The advantage of cementation is that it is independent of the axial alignment of the implants. This is often indispensable for crowns and bridges. Furthermore, a loosening of the screw connection cannot lead to a fracture or loss of the implant screw [1, 2]. However, the screw channel represents a weak point in terms of the material stability of the crown and its cleanability. Aesthetic limitations caused by any visible screw access may be eliminated with cementation [3] and a better framework fit has been described [1, 2, 4].

One of the advantages of screw fixation concern die peri-implant tissue [5]. Thoma et al. found a lower pathogenic bacterial spectrum around screw-retained crowns than around cemented crowns on implants [4]. Furthermore, if the abutment screw becomes loose, it can be tightened again without any problems [6, 7]. In general, accessing and loosening or reattaching the implant-supported restoration is very easy with this option [2, 6, 8, 9].

Cemented restorations can be made retrievable by the use of semipermanent cements [10] which has become popular in recent years. Temporary cements have been successfully used for metal-ceramic restorations on titanium abutments for many years.

For semipermanent fixation on implants, it is required that the retention forces are in an area which, on the one hand, ensures undisturbed function under masticatory load and, at the same time, is stable in the aqueous environment of the oral cavity. On the other hand, it should allow the superstructure to be released when required without endangering the implant [11, 12]. Some authors have considered retention forces in the range of 50-200 N to be necessary for this purpose [13–17]. If the superstructure is removed for whatever reason, cement residues remain on the abutment and in the crown lumen. These cement residues should be easily removable in order to avoid damaging either the abutment or the crown. A statement about the removability of cement residues could be derived from the fracture mode.

For this study, three established cements (Harvard Implant Semi-permanent®, implantlink® semi Forte and Temp Bond® NE) were selected and compared with respect to their retention force. The aim of the present study was to examine whether the retention of these cements is comparable for the temporary cementation of monolithic zirconia superstructures. Furthermore, a fracture mode analysis should allow conclusions to be drawn about the removability of cement residues.

The following null hypotheses were tested:

1. The pull-off forces of the three temporary cements Harvard Implant Semi-permanent (HAV), implantlink semi Forte (IMP) and Temp Bond NE (TBNE) do not differ.

2. The fracture modes of the three temporary cements HAV, IMP and TBNE after removal do not differ.

## Methods

**Fig. 1:**
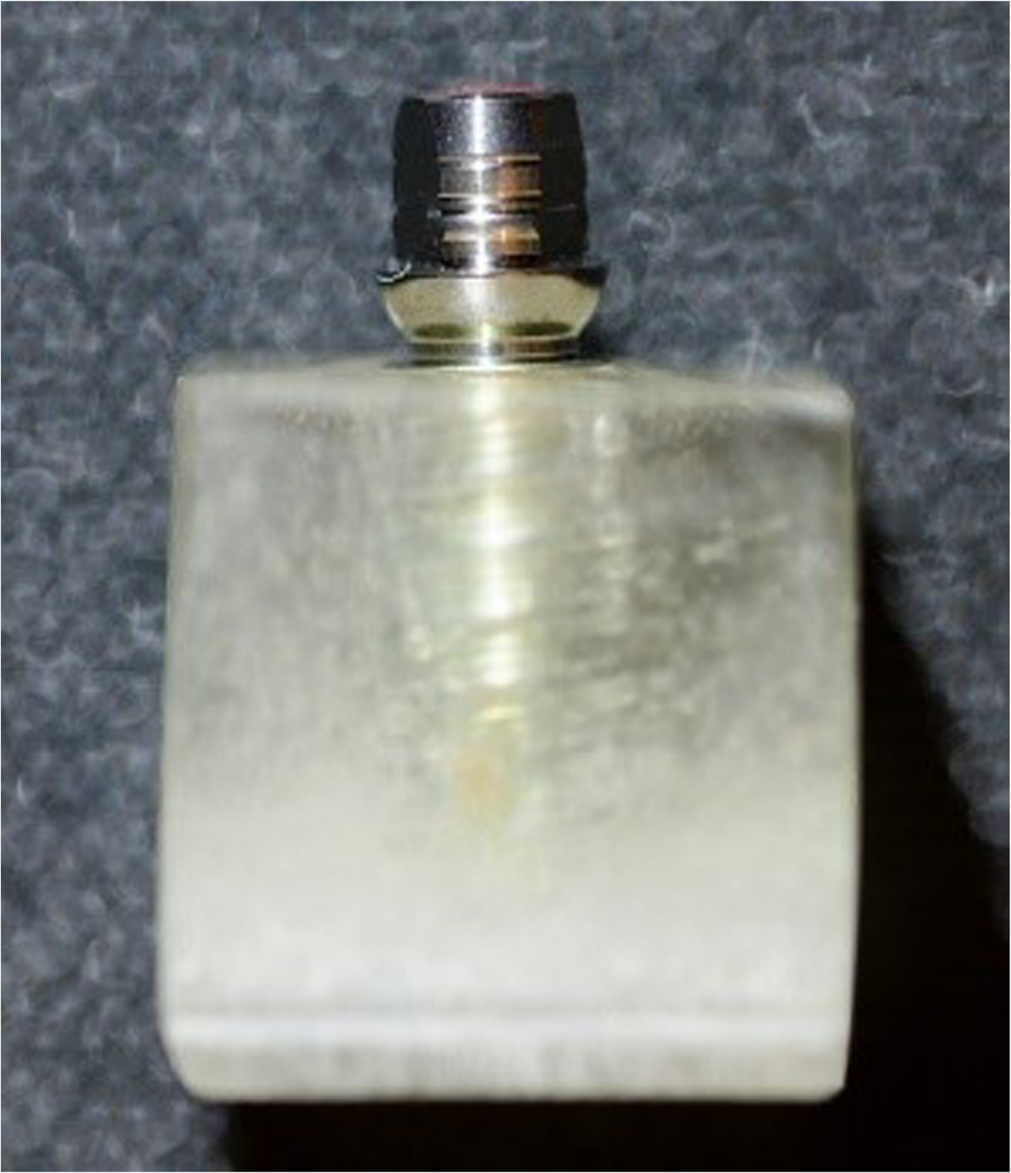
Side view of an embedded specimen

In the study, 20 test specimens were produced per cement, of which 10 test specimens each were subjected to thermocycling. The number of samples was chosen based on other studies that addressed this issue [18–21]. Sixty implants (iSy®, CAMLOG, Wimsheim, Germany) based on titanium with a length of 11 mm and diameters of 4.4 mm were used. These were pre-screwed with Camlog iSy® titanium abutments (Camlog, Wimsheim, Germany) of the following geometry: diameter 4.0 mm, prosthetic height 4.6 mm, gingival height 1.6 mm and a cone angle of 3° (Fig. 1). The cement gap size was set at 30 μm.

**Fig. 2:**
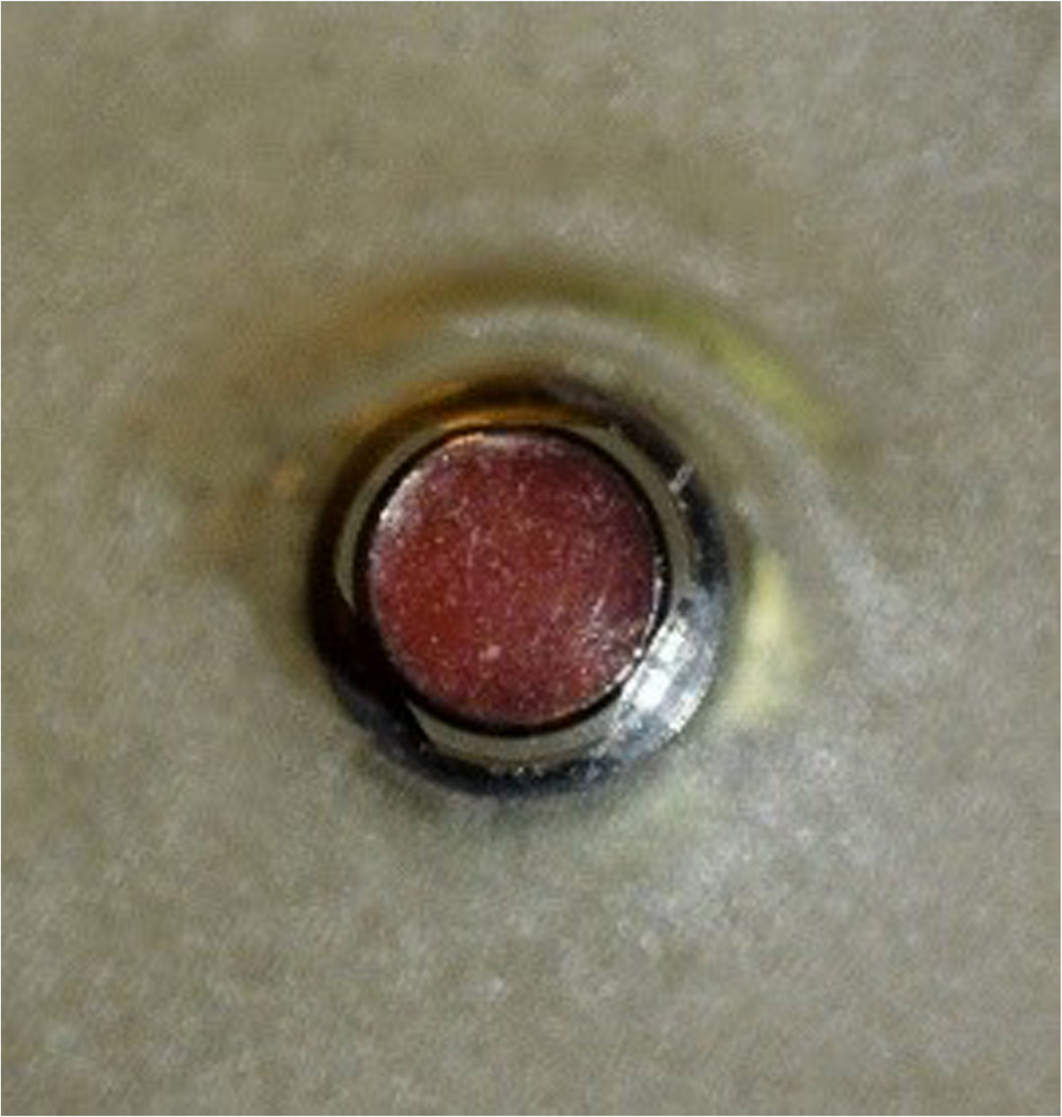
Top view of the abutment with rotation lock

The abutments were provided with two horizontal circumferential grooves. Furthermore, the abutments were designed having three rotation lock areas to generate a rotation protection (Fig. 2).

**Fig. 3:**
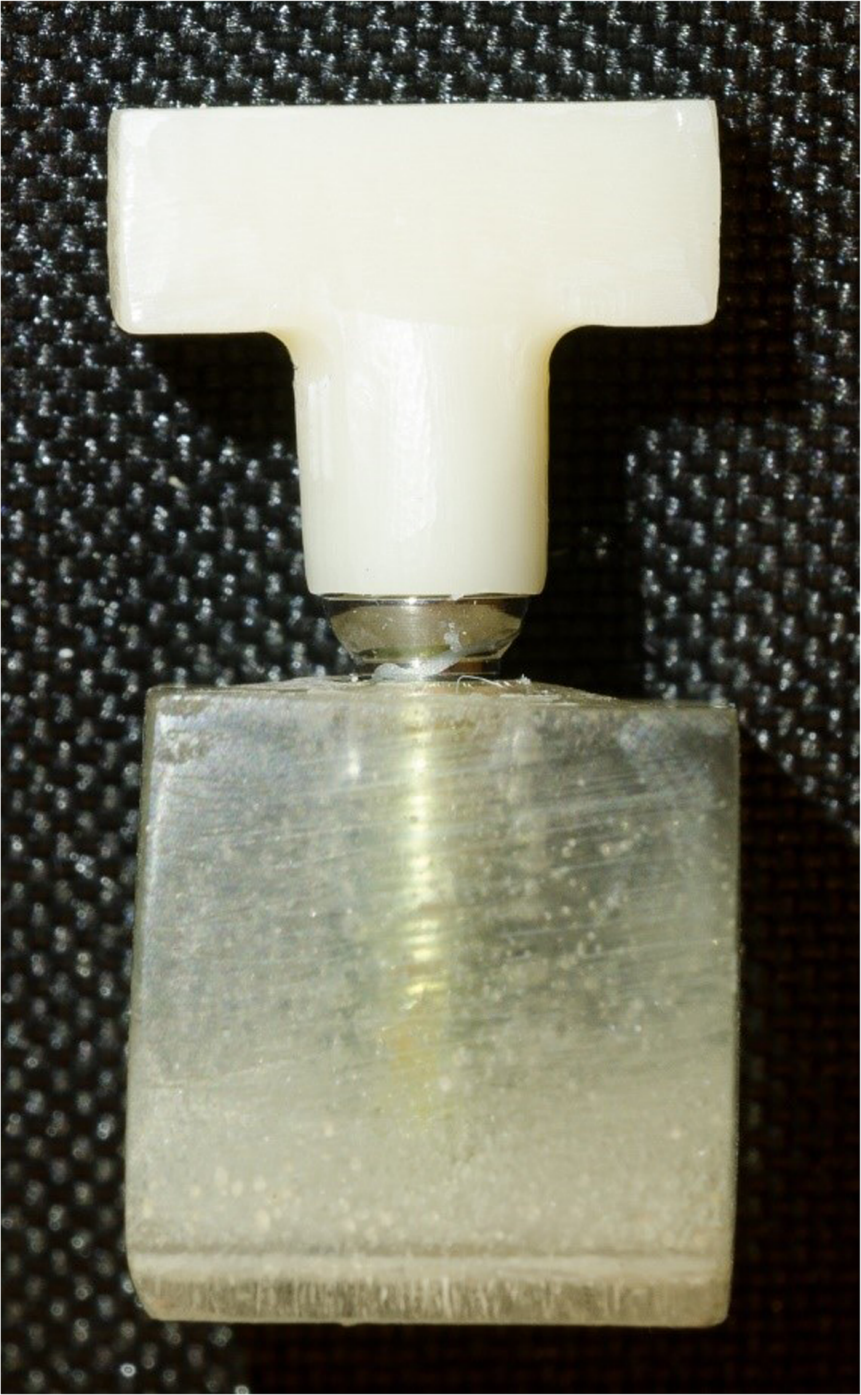
Embedded implant with cemented superstructure

All screw channels were closed with wax. Subsequently, the implants were embedded in a two-component resin (Paladur transparent, Kulzer, Hanau, Germany) (Fig. 3).

To fabricate the implant superstructure, the implant abutments were scanned with a desktop scanner (D900L, 3Shape A/S, Copenhagen, Denmark). With the help of a design software (Dental System Premium, 3Shape A/S, Copenhagen, Denmark), planning of the superstructure (wall thickness 0.5 mm, cement film thickness 30 μm) was carried out in the form of a test specimen with lateral retaining wings (length 3 mm, width 3 mm, height 4 mm) for subsequent clamping in the retaining bracket. The test specimens were milled from highly translucent zirconia discs (DD Bio ZX^2^ colour 98, Dental Direkt, Spenge, Germany) and then sintered at 1450 °C with a holding time of 120 min. Subsequently, the test specimens were randomly distributed to three main groups (A-C).

*Group A*. Harvard Implant Semi-permanent®: a dual-curing semipermanent cement based on multifunctional methacrylates and zinc.

*Group B*. implantlink® semi Forte: a dual-curing semipermanent cement based on meth- and urethane acrylate.

*Group C*. Temp Bond® NE: a eugenol-free semipermanent cement based on zinc oxide.

**Fig. 4:**
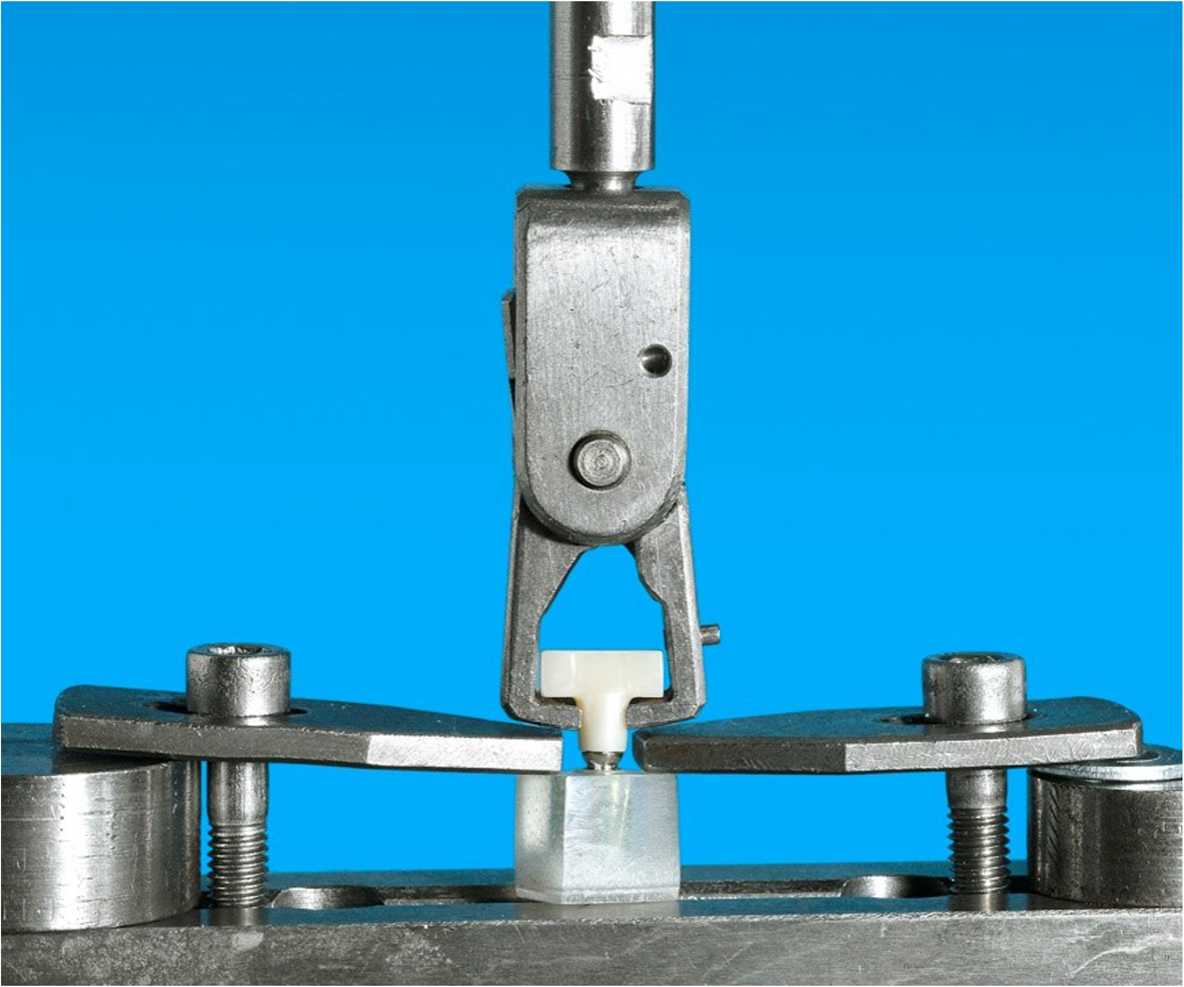
Clamped test specimen with retaining clips positioned under the retaining wings

All implant abutments and the interior of the test specimens were cleaned with ethanol (70%) before cementation and then dried with oil-free compressed air. Afterwards, the crown lumina of the test specimens were coated with a small amount of the respective temporary cement using a disposable brush and pressed onto the implant abutment as close as possible to the final position (= abutment shoulder). Immediately afterwards, the superstructure was pressure loaded with 35 N for 30 s in the universal testing machine TIRAtest 2720 (TIRA, Schalkau, Germany). The specimens were then stored at room temperature for 30 min until the complete setting reaction had occurred. After careful removal of excess cement residues, the specimens were stored for 24 h in distilled water at 37 °C. Half of the test specimens of each group were then subjected to thermocycling (TC) of 6000 cycles (5 °C/55 °C) with an exposure time of 27 s per basin. The retention forces were determined with the universal testing machine TIRAtest 2720 (TIRA GmbH, Schalkau, Germany). For this purpose, the resin blocks with the specimens embedded at a 90° angle were fixed centrally in the platform of the testing machine with two opposing retaining wings. A holding bracket, which was attached centrally to the beam with the force sensor, was positioned under the holding wings of the specimens (Fig. 4). At a test speed of 1 mm/min, an axial tensile load was applied via the retaining wings until failure of the tension. The pull-off force was derived via the integrated load cell and recorded in a force-path diagram. Immediately after completion of the pull-off force measurement, the clamped test specimens were removed and classified with regards to their fracture modes with the aid of magnifying glasses at ×2.5 magnification (Table 1, Fig. 7). Statistical significance was determined by means of the single-factor analysis of variance according to Kruskal-Wallis and the post hoc test according to Dunn-Bonferroni. The chi-square test of independence was used to compare breaking modes for temporary cements with and without TC.

**Table 1:** Classification of the breaking modes and their characteristics

Type	Breaking modes	Characteristics
I	Adhesive cement fracture on the abutment	The abutment is largely cementless. The cement is completely in the crown lumen.
II	Adhesive/cohesive cement fracture on the abutment	There are cement residues on the abutment but not to an extent of the full thickness of the cement film. The majority of the cement is located inside the crown.
III	Cohesive cement fracture	The abutment and the crown lumen are mainly covered with cement. The separation takes place within the cement film.
IV	Adhesive cement fracture at the crown	The crown lumen is largely free of cement. The cement is completely on the abutment.
V	Adhesive/cohesive cement fracture at the crown	In the crown lumen are cement residues but not the full thickness of the cement layer. The majority of the cement is on the abutment.

## Results

**Fig. 5:**
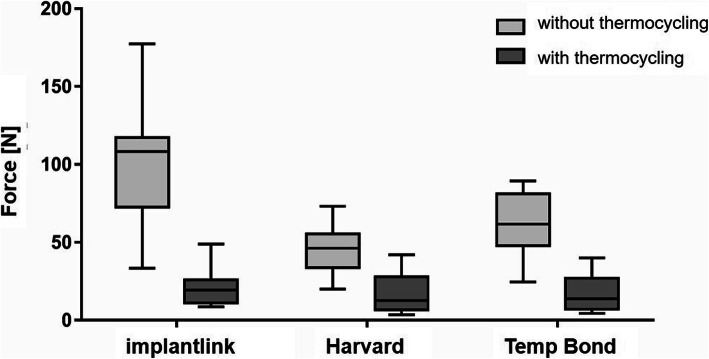
Decementation load for the three tested cements (mean values with standard deviation)

Without TC, the retention-force measurements (Table 2, Fig. 5) of the temporary cements showed significant differences between the groups (*p* = 0.0003 in one-way ANOVA). The highest mean pull-off forces (100.5 ± 39.14 N) were measured for IMP and the lowest mean pull-off forces (45.78 ± 15.66 N) for HAV (*p* < 0.001; Bonferroni’s multiple comparisons test; IMP vs. HAV). The measured pull-off forces of TBNE of 61.16 ± 20.19 N ranged between the average pull-off forces of the other two cements (*p* <0.01; Bonferroni’s multiple comparisons test; IMP vs. TBNE). After TC, the pull-off forces of the three tested temporary cements were reduced. The pull-off forces measured for IMP were highest at 21.69 ± 13.61 N (three fail outs). HAV showed pull-off forces of 17.38 ± 12.77 N, similar to TBNE with 16.97 ± 12.36 N (two fail outs). Statistically significant results could not be demonstrated.

**Table 2:** Pull-off forces in Newton (N) for each temporary cement per specimen before and after TC, fail outs were observed for Implantlink and Temp Bond NE with TC

Implantlink (without TC)	Harvard (without TC)	Temp Bond NE (without TC)	Implantlink (with TC)	Harvard (with TC)	Temp Bond NE (with TC)
112.47	19.95	53.81	19.3	5.18	10.9
78.18	42.15	50.74	48.9	14.1	9.52
177.26	54.24	24.6	23.9	9.03	16.5
109.52	53.97	89.34	11.1	27.6	30.1
116.35	36.31	67.03	14.1	11.2	40.1
106.89	26.51	82.89	8.56	42	4.47
33.4	59.05	58.12	26	28.5	6.36
119.58	73.04	80.43		7.14	17.8
55.94	44.49	39.42		3.48	
95.25	48.12	65.25		25.6

**Fig. 6:**
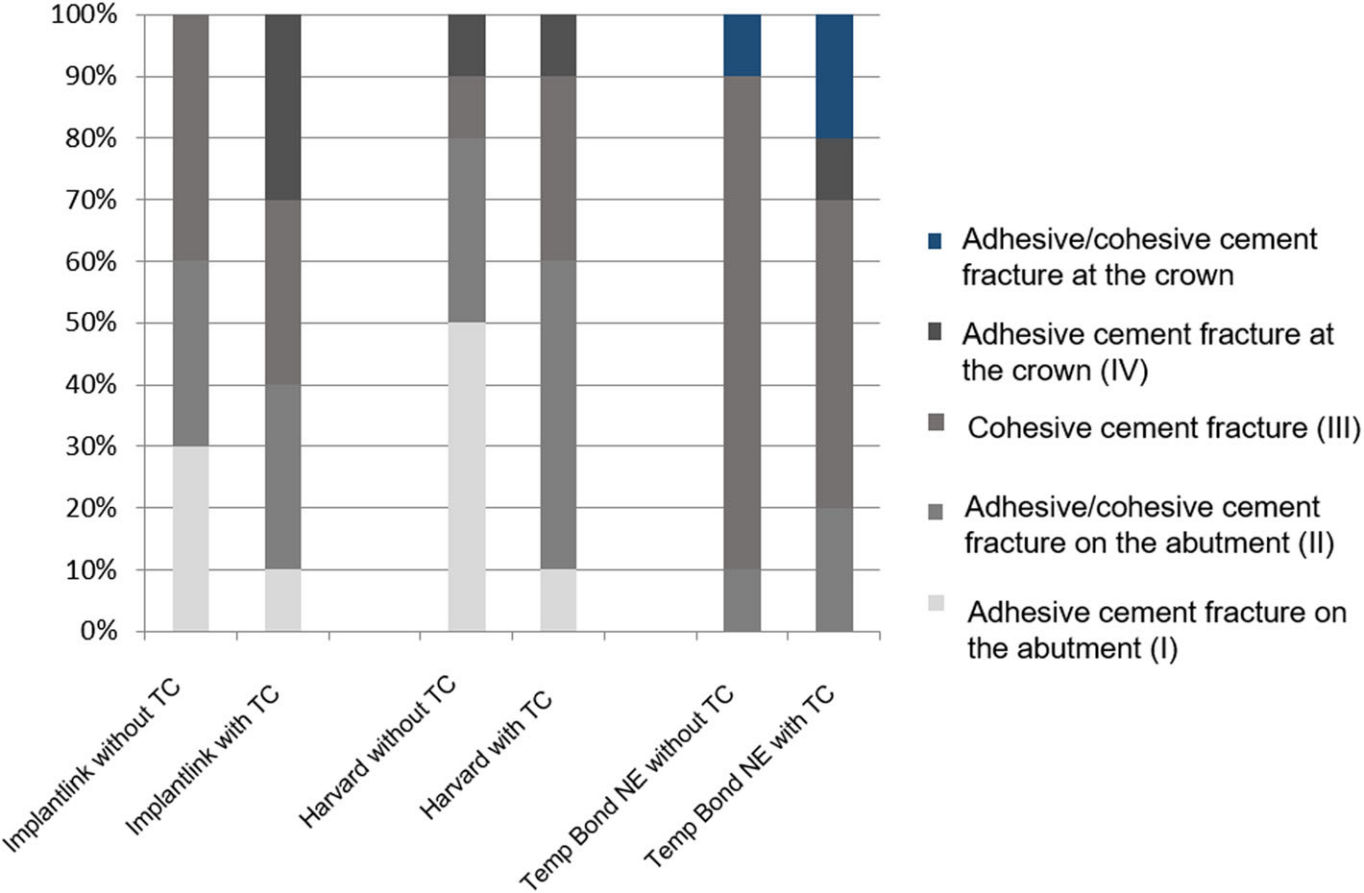
Fracture mode analysis of the tested temporary cements

Specimens of the HAV group without TC predominantly showed type I fractures (Table 3, Fig. 6). This fracture type was characterised by individual cement residues on the abutment. Type II fractures of this group were characterised by small cement residues in the area of the abutment shoulder. Types III and IV fractures were each represented once. The same four fracture types were also identified in the group with TC (Table 3) (*p* = 0.2440 in chi-square test of independence without vs. with TC).

**Table 3:** Number of specimens regarding the fracture mode for each temporary cement

Cement	Adhesive cement fracture on the abutment (I)	Adhesive/cohesive cement fracture on the abutment (II)	Cohesive cement fracture (III)	Adhesive cement fracture at the crown (IV)	Adhesive/cohesive cement fracture at the crown (V)
**Implantlink without TC**	3	3	4	0	0
**Implantlink with TC**	1	3	3	0	0
**Harvard without TC**	5	3	1	1	0
**Harvard with TC**	1	5	3	1	0
**Temp Bond NE without TC**	0	1	8	0	1
**Temp Bond NE with TC**	0	1	5	1	1

TBNE specimens without TC showed mainly type III fractures (Table 3, Fig. 6). These showed differently imprinted cement residues on the abutment and inside the superstructure. Types II and V fractures were detected once each. The specimens with TC showed four fracture types (Table 3, Fig. 6) (*p* = 0.6849 in chi-square test of independence without vs. with TC). Five type III fractures represent the most common fracture type. Types II, IV and V fractures were detected once each (Table 3, Fig. 6). For type V fractures, it is typical to find cement residues in the crown lumen, but the main part of the cement is on the abutment.

**Fig. 7:**
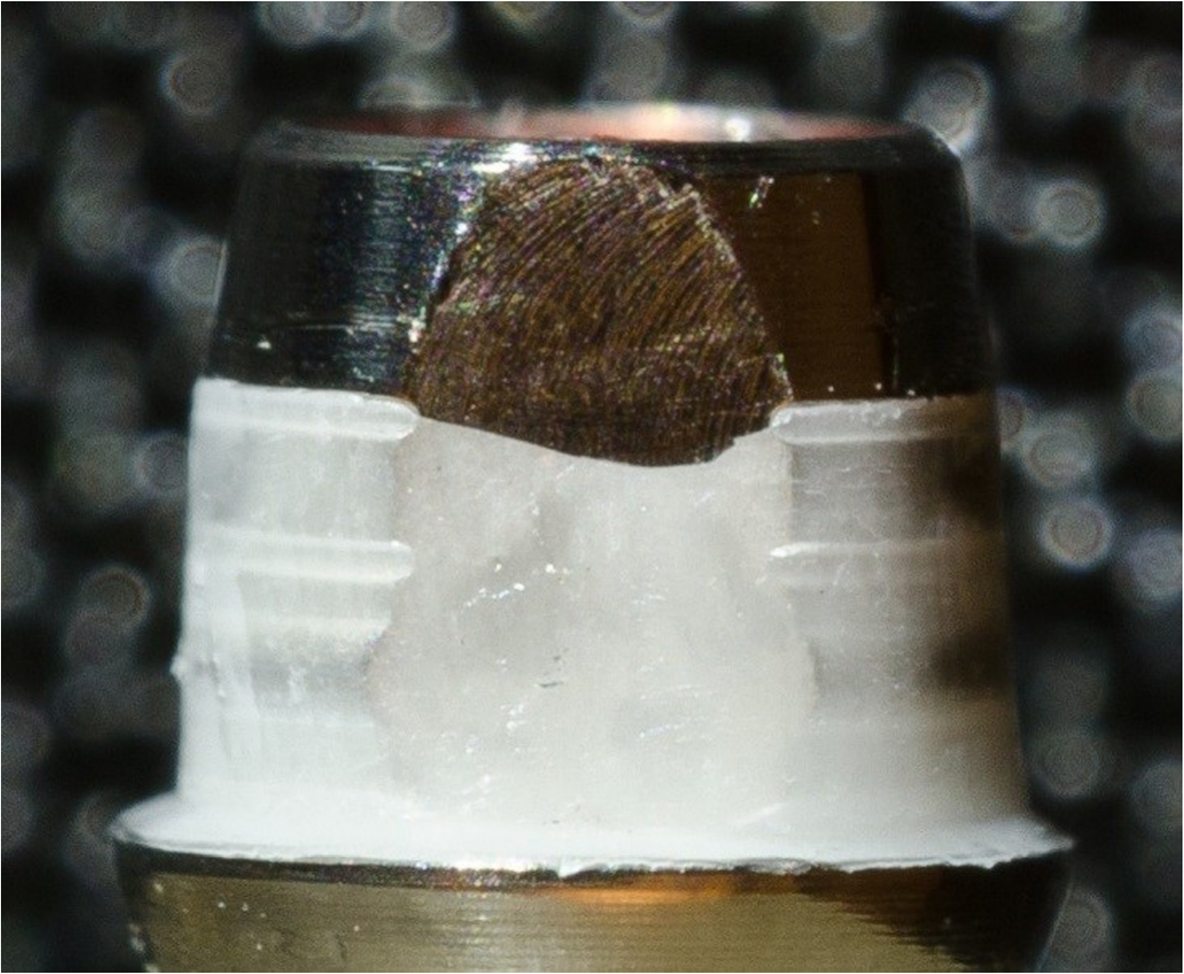
Cohesive cement fracture (type III; implantlink semi Forte)

Without TC, the fracture mode analysis for IMP showed a similar distribution of fracture types I, II and III (Fig. 7). After TC, IMP showed a reduction of types I and III fractures (*p* = 0.7286 in chi-square test of independence without vs. with TC) (Table 3, Fig. 6).

## Discussion

In the present study, retention forces for the three tested temporary cements differed. Therefore, the first null hypothesis must be rejected since the objective of this study was to demonstrate homogeneous retention values of the three cements tested. Other studies also showed heterogeneous results regarding the retentive strength of different cements [5, 21–39]. Therefore, some studies published guidelines for clinicians since no cement served for all demands [40, 41]. Furthermore the retentive strength was very heterogeneous in the individual material classes and therefore not comparable [40].

The fracture modes of the three tested temporary cements after TC differ in the present study. As a consequence, the second null hypothesis must be rejected as well. The chi-square test did not show significant correlations. The fracture mode allows one to draw conclusions about the removability of cement residues when crown removal is necessary or when the crown detaches itself and re-cementing is required [42]. However, the fracture mode analysis provides information that influences the cement selection [42]. Cement residues in the crown lumen are easily removed by blasting with Al_2_O_3_ and improve the retention of the superstructure [10, 43, 44]. Therefore, fractures of types I and II, which were predominantly detected at IMP and HAV, can be assessed as positive [42]. In these fracture types, the majority of the cement is in the crown lumen, and the abutment is largely free of cement. The screw access in the abutment is free, and the abutment is not further damaged when trying to remove cement residues [42].

To bring the results closer to clinical conditions, an artificial ageing process was conducted. Test specimens were stored for 24 h in distilled water and half of the test specimens of each group were subjected to a thermocycling process. The number of cycles was chosen based on other studies that have addressed this issue [21, 23, 45]. Gale and Darvell proposed that 10,000 cycles might represent approximately 1 year of in vivo conditions, with 20 to 50 cycles considered equivalent to a single day [46]. It can be concluded that 6000 cycles correspond to about 7 months of clinical use. The water storage was used to simulate oral cavity moisture. During water storage, moisture absorption occurs in the cement gap, which causes an increase in volume of the cement [37, 38]. In addition, water storage leads to a wash-out effect within the cement structure and thus simulates the ageing process of the cements in the oral cavity [5, 10, 37]. This artificial ageing can be enhanced by TC and influences the retention forces of the different cements very differently depending on the number of cycles [5, 37, 38]. Thermocycling is an artificial ageing in vitro and imitate temperature changes as they occur naturally intraorally. However, thermocycling can be used to mimic the clinical situation, but it has no relevance for direct comparisons to in vivo conditions [19]. Some studies have performed thermocycling in order to evaluate retention forces of the cements tested [5, 20–38]. For temporary cements, a considerable decrease in regards to the pull-off forces has been proven to be caused by the artificial ageing process [22]. A direct transfer of the results obtained from the present in vitro study to the clinical situation should be viewed critically.

Regarding the abutment geometry, significantly shorter abutments (3 mm) were used in this study compared to other studies regarding this topic [10, 47]. However, abutment heights of 5.5 mm or more are not always clinically feasible. Despite the significantly lower abutment height, the measured retention forces of the three cements were in a range that the cements also showed in other publications [5, 48]. Results by Safari et al. already showed that the abutment height has less influence on retention than the abutment geometry [42]. In contrast, Abbo et al. showed that a reduction of the abutment height by 1 mm significantly reduces the retention force [49]. This was also confirmed by Sarfaraz et al. [50]. Lewinstein et al. reported that the retention forces of temporary cemented crowns on implant abutments can be significantly increased by circumferential grooves [29]. The abutments used in the present study were provided with two grooves. Pull-off forces showed values as published by other working groups despite the low abutment height of 3 mm [48].

In the present study, the cement gap size was set to 30 μm. So far, mostly different cement film thicknesses have been compared in different studies [34, 40, 51–59]. Ebert et al. showed that increasing the cement gap from 30 to 60 μm had a detrimental effect on the durability of cements [56]. On the other hand, Wu and Wilson showed that for optimal seating, the cement gap should be larger than 30 μm [60]. Mehl et al. showed that the pull-off force of implant-supported crowns is significantly worsened by an increase of the cement gap from 10 to 50 μm [2]. Some authors assessed low cement gap thicknesses as an advantage in terms of pull-off forces [2, 61]. Most studies recommended a cement thickness of 20 to 40 μm, as this size generates complete seating of the restoration [62–65]. Gultekin showed that increasing the cement gap from 20 to 40 μm for lower strength cements such as TBNE did not significantly increase retention [40]. For this reason, a cement gap of 30 μm was selected in the present study, in which semipermanent cements were investigated with regard to their retentive strength.

From the generated data, it can be concluded that cements specially developed for the temporary cementation of superstructures on implant abutments do not differ from the conventional temporary cement TBNE. The risk of decementation seems to increase with temporary cements. Therefore, temporary cementation of zirconia crowns on titanium abutments is recommended reluctantly. However, cements that have been specially developed for temporary cementation on implant abutments are more likely to be used when retrievability of the superstructure is considered likely.

## Conclusions

The present study showed that the influence of artificial ageing is important for imitating clinical conditions regarding retention forces. IMP showed the highest pull-off forces under the chosen test conditions. All three temporary luting agents showed significantly lower retention forces after thermocycling. Because of the easy cement removal in the crown lumen, the dominance of adhesive cement fractures on the abutment and adhesive/cohesive cement fractures on the abutment with HAV appear to be an advantage in case of recementation of the superstructure and thus gives clinicians advice which cement to use for different requirements.

## Data Availability

All data generated or analysed during this study are included in this published article (and its supplementary information files).

## Abbreviations

HAV: Harvard Implant Semi-permanent
IMP: Implantlink semi Forte
TBNE: Temp Bond NE
TC: Thermocycling
